# Monitoring the Effectiveness of Emergent Detached Offshore Structures in Mangrove Vegetation Increase: Lessons and Recommendations

**DOI:** 10.3390/life15020136

**Published:** 2025-01-21

**Authors:** Nguyen Tan Phong, Nguyen Bao Thuan, Nguyen Ngoc Tien

**Affiliations:** 1 Faculty of Environment and Labour Safety, Ton Duc Thang University, Ho Chi Minh City, Vietnam; baothuan.btcmep@gmail.com; 2 Institute of Marine Geology and Geophysics, Vietnam Academy of Science and Technology, Hanoi, Vietnam; nntien@imgg.vast.vn

**Keywords:** natural regeneration, offshore breakwaters, stabilization process, secondary succession, vegetation cover

## Abstract

Although successful in protecting planted mangrove plants, the effectiveness of emergent detached offshore structures in increasing vegetation cover has yet to be definitively determined. We selected Tien Giang Province, Vietnam as an appropriate case study to address this question. We analyzed multiyear (2000 and 2022) shoreline changes and calculated the enhanced vegetation index (EVI) together with ground truthing in pursuit of the objectives of the study. Our findings suggest that emergent detached offshore structures have yet to lead to an increase in vegetation cover or promote mangrove growth. The vegetation growth steadily increased, as did the high level of natural mangrove growth with fully grown mangrove trees, even before the structures were constructed. By 2015, all the categories increased slightly except for low vegetation cover (LVC) and medium vegetation cover (MVC). LVC decreased from 390 ha in 2010 to 291 ha in 2015, while MVC decreased from 305 ha in 2010 to 275 ha in 2015. By 2020, all the categories decreased slightly except for non-vegetation cover—Barren lands (NVC2) and MVC. NVC2 decreased slightly from 404 ha in 2015 to 368 ha in 2015. The MVC decreased slightly from 275 ha in 2015 to 212 ha in 2020. Non-vegetation cover—Intertidal mudflats (NVC1)—LVC, and high vegetation cover (HVC) increased slightly from 2015 (326 ha, 291 ha, and 249 ha, respectively) to 2020 (368 ha, 292 ha, and 298 ha, respectively). By 2022, NVC2, MVC, and HVC remained unchanged, while NVC1 and LVC increased slightly from 368 ha and 292 ha in 2015, respectively, to 380 ha and 302 ha, respectively. The increase in vegetation cover and the natural regeneration of mangrove species were partly due to the adaptation of mangrove species to the site (river mouth areas), particularly the protection provided by Ngang Island offshore, and the construction of these structures. In addition, these structures were constructed in a rather stable area (slightly eroded and estuarine area) and therefore have yet to provide any noticeable benefits for mangrove regeneration three to five years after their construction. In the future, the morpho dynamic and hydrodynamic elements of the site should be adequately considered during the design and construction of these structures to increase vegetation cover and promote natural mangrove regeneration.

## 1. Introduction

Offshore breakwaters are structures constructed parallel to the shore in more exposed settings or deeper water areas at a certain distance from the shoreline. These structures are constructed primarily to dissipate the energy of incident waves [1]. Offshore breakwaters can be emergent detached structures [1] or low-crested/submerged structures [2]. Emergent detached structures are normally constructed with gaps among structures to facilitate water exchange and sediment transport. Low-crest or submergent structures are constructed in a long continuous form without gaps, to reduce the intensity of wave action and promote water circulation. To date, emergent detached offshore structures have been constructed to protect sandy beaches from wave-induced erosion at various locations around the world; for example, in Italy [3,4], the Mediterranean basin [5], and the UK [6]. To date, a numerical test suggested that the emergent breakwaters constructed in Italy and European Union countries should be replaced by submerged breakwaters to avoid the increment of wave transmission, to complete full dissipation of the wave energy, and to reach transmission coefficient [1]. A practical lesson in Italy showed that the conversion of offshore breakwaters into continuous submerged breakwaters provided better views of the sea and a more equitable beach width along the shore, allowing natural processes to reshape the beach [7]. Gaps between these structures and the shoreline, wave angle, and wave height are crucial in determining sediment accumulation ratios [8].

Similar structures have increasingly been used to respond to the erosion of mangrove forests in some areas of Southeast Asia. While these structures were constructed in combination with mangrove planting in Malaysia [9,10,11], mangrove planting was not undertaken with these offshore structures in Vietnam [12,13]. To date, these offshore concrete breakwaters have been successful in dissipating the energy of incident waves and protecting planted mangrove seedlings but accumulated less fine-grained sediment in Malaysia [10,11]. Submerged offshore structures, although costly, have been effective at protecting planted mangroves in some areas of Southeast Asia; for example, Indonesia [14] and Vietnam [15]. Against this background, it remains unclear whether offshore breakwaters are able to facilitate mangrove growth. This knowledge is crucial, particularly because offshore structures have been promoted as effective solutions to the problem of coastal erosion and for improved adaptation to the adverse effects of future climate change.

The Vietnamese Mekong Delta (VMD) has been severely eroded, with approximately 45% of the VMD coast being severely eroded and a maximum erosion rate of 45 m per year [16]. As the VMD plays a crucial role in Vietnam’s social–economic development, the Vietnamese government has made significant efforts to control coastal erosion in this region [17]. In 2013, Ca Mau province (one of the VMD provinces) was the first one in the region to have invested in constructing offshore breakwaters for constructing its coast [15]. This construction was based on the belief that strong incident waves were a driving force that had eroded muddy coasts [18]. In 2021, these offshore breakwaters were successful in dissipating the energy of strong incident waves and protecting stands of mangrove trees [15]. Given the success case of the Ca Mau offshore breakwaters [15], and that fact that other coastal protection measures such as the planting of mangrove seedlings and sea dykes have had limited success in protecting these coasts [18], multiple concrete (shoreline and offshore) structures have been strongly recommended to protect this coast in order to maximize the effectiveness of these offshore structures in protecting eroding coasts [19]. In 2019, Tien Giang province was the second one of the VMD to construct emergent detached offshore structures, called hollow triangular offshore breakwaters (HTBs), along the coast of the Tan Phu Dong district, Tien Giang Province, Vietnam [12,13]. By 2023, the coast of Tan Phu Dong District experienced an increase in vegetation cover and the significant natural regeneration of mangrove species (*Sonneratia*, *Avicennia* and *Bruguiera* sp.). It was reported that Tien Giang HTBs facilitated mangrove restoration and increased vegetation cover in the shoreside area of the Tan Phu Dong district, Tien Giang Province, Vietnam [12,13]. In 2021, the Tien Giang Provincial level People’s Committee (Tien Giang PPC) replicated this structural design in other areas along the Tan Thanh and Tan Dien coasts of Tien Giang province (a total of 3 km of HTBs), with funding from the local and central governments. Other provinces have expressed their interest in replicating similar structures to protect their eroding coasts. Governments at all levels also considered the Tien Giang HTBs as a success case in the region. However, little information is available on why and how vegetation cover has increased in the area.

Therefore, Tien Giang HTBs were selected as an appropriate case study to partially address the question raised in the literature and the reality. This study aimed to investigate the relationship between Tien Giang HTBs and vegetation cover increase. Our primary objectives were to investigate (1) how vegetation cover increased behind and around HTBs, and (2) whether the location of Tien Giang HTBs contributed to the increase in vegetation cover. The first objective was achieved by calculating the enhanced vegetation index (EVI) of the entire coast of the Tan Phu Dong district of Tien Giang Province, including the vegetation behind Tien Giang HTBs and field observations. The second one was obtained from the analysis of shoreline change using digital shoreline change analysis (DSAS)—version 5.0. DSAS is an extension of ArcGIS 10.8. software.

## 2. Materials and Methods

### 2.1. Site Description

**Figure 1:**
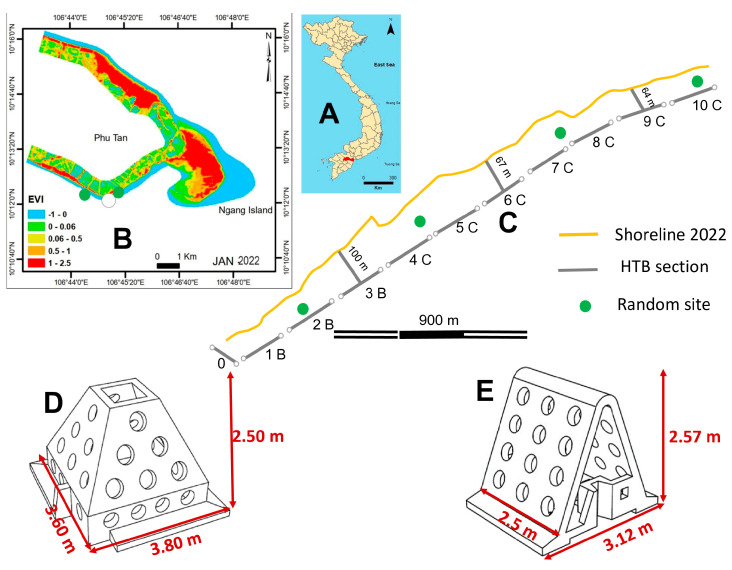
The location of the study site and the technical design of Tien Giang HTBs. (**A**) The location of Tien Giang Province in Vietnam (red color). (**B**) The 2022 EVI map, with the white dot indicating the study site and the two green dots indicating two sites outside the HTBs as the control sites. (**C**) The study profile with the Tien Giang HTB deployment and four random sites (green dots). (**D**) The technical design of Tien Giang HTB—Type A. (**E**) The technical design of HTB—Type B (adapted from previous studies in the area [12,13]). (See Table 2 for further information on technical design of Tien Giang HTBs and their length and types).

Go Cong is located in the Tan Phu Dong district, Tien Giang, Vietnam. Hydrologically, the study site is strongly influenced by the southwest and northeast monsoon regimes. The southwest regime starts from May to October (the rainy season) while the northeast monsoon regime runs from November to March (the dry season). Tien Giang is substantially influenced by the East Sea tidal regime, which features semidiurnal tides and diurnal inequality. The tidal range varies between +1.8 and +2.2 m [20]. Located in the estuarine area of the Dai River, the site is dominated by mangrove species common in estuarine areas of rivers and tidal riverbanks, including *Sonneratia alba*, *Sonneratia caseolaris*, *Avicennia marina*, and *Bruguiera cylindrica* (Figure 1).

By 2019, HTBs were designed and constructed by the Southern Institute of Water Resources. HTBs have a dense porosity designed to maximize wave transmission, reflection, and dissipation. HTBs were numerically tested using glass wave flumes equipped with a pointed-trapezoidal cross-section and with two sides containing circular holes in four rows of variable diameter to represent different scenarios [12]. Unlike other impermeable structures, HTBs were designed with circular holes to reduce reflected waves and ensure the exchange of water, sediment, and water quality [12]. Wave gauges were installed in front of and behind the structures that were placed in the flumes to measure wave transmission and incident and to reflect wave parameters separately [12]. Numerical modeling revealed that HTBs were effective at dissipating incident wave energy [12]. HTBs were subsequently constructed to protect a tourist site in Go Cong, Tan Phu Dong District, Tien Giang Province in 2019 (Tien Giang HTBs) [12] for a total length of 1.6 km. The design and construction of the study were funded by the Vietnamese National Research Program. Tien Giang HTBs had been constructed for three years at the site (Figure 1D,E). The construction aimed to dissipate the energy of strong incoming waves, accumulate fine-grained sediment, and promote mangrove growth. Therefore, mangrove plants or propagules were not planted behind Tien Giang HTBs.

### 2.2. Methods

This study involved two phases, including EVI calculation and DSAS. The EVI calculation was undertaken in this study because the use of the Normalized Difference Vegetation Index (NDVI) is strongly affected by the brightness of the ground [21] and the saturation in high biomass areas [22]. The EVI calculation only aimed to record vegetation cover for the purpose of the study, not to identify mangrove species.

To calculate EVI values, the authors retrieved five satellite images between 2000 and 2022 from the United States Geological Survey (https://glovis.usgs.gov/) (accessed on 18 December 2023). The authors selected 2000 as the starting year for the analysis because erosion along the Tien Giang coast was first identified at this time. The 2022 satellite image was selected to update the status of vegetation cover and the HTBs. The authors used two rules for selecting satellite images: (1) the images that covered the study area were selected and (2) the images with an average cloud cover lower than 20% were selected. Each image was clipped to ensure that only the study area’s pixels were retained (Table 1).

The authors strictly followed the previous recommendation on defining the land–water boundary (yearly shoreline) [23,24,25,26] to avoid possible errors when calculating vegetation cover. This is particularly true for areas which are likely subject to inundation due to seasonal tidal regimes and for satellite images retrieved on different dates.

Yearly shorelines were established using three steps: (1) calculation of the modified normalized difference water index (mNDWI), (2) calculation of the water frequency index (WFI), and (3) establishment of yearly land–water shapefiles.

The *mNDWI* was calculated using the following Formula (1):

(1) $$mNDWI=\frac{Green-MIR}{Green+MIR}$$

where

*Green* = Green band

*MIR* = Mid-infrared radiation

The *mNDWI* value ranges between −1 and 1, where positive values represent pixels with water.

The *WFI* was calculated using the following Formula (2):

(2) $$WFI=\frac{N_{water}}{{N_{water}+N}_{land\;}}$$

where

*N_water_* and *N_land_* denote the number of pixels that were observed as water and land within 1 year, respectively.

*WFI* pixel values greater than or equal to 0.5 (equivalent to a frequency of 50%) were reclassified as representative of the annual water/surface area.

Satellite images from different dates (2000, 2005, 2010, 2015, 2020, and 2022) were digitalized to extract multiple date satellites as vector ‘shapefiles’ using ArcGIS 10.6 software. These ‘shapefile’ sectors were overlaid to detect changes in the shoreline and the vegetation cover between 2000 and 2022.

The *EVI* formula is as follows (3):

(3) $$EVI=\frac{2.5\times \left(NIR-R\right)}{NIR+(2.4\times R)+1.0}$$

where

*IR* is the near-infrared band (Band 5—Landsat 8; Band 4—Landsat 7)

*R* is the red band (Band 4—Landsat 8; Band 3—Landsat 7)

We classified Go Cong, Tien Giang Province, Vietnam into two classes: vegetated areas (mangrove forests) and non-vegetated areas (intertidal mudflats, barren lands, and aquaculture ponds). In addition, overall accuracy (*OA*) and Kappa coefficient (KC) were used to evaluate the accuracy of the extraction results.

The *OA* formula is as follows (4):

(4) $$OA=\frac{TP+TN}{T}\times 100\%$$

where

*TP* (True Positive) and *TN* (True Negative) represent, respectively, water and non-water pixels/points that match with the reference sites

The *KC* formula is as follows (5):

(5) $$KC=\frac{\left(TS\times TCS\right)-Ʃ(Column\;Total\times Row\;Total)}{{TS}^{2}-Ʃ(Column\;Total-Row\;Total)}$$

where

*TS* = Total sample

*TCS* = Total corrected sample

The evaluation showed that the OA was 92% and the KC was 0.90, indicating that the proposed methods used in this study agreed with the previous recommendations that the maximum OA value is up to 100% and the maximum KC value is up to 1, as recommended previously [27,28].

The authors used the DSAS method for identifying shoreline change in Tien Giang Province, as previously recommended [23,25,29,30,31,32]. To commence the DSAS, a baseline was established as a curved line located 300 m landward over 12 km, stretching from the north to the south of Tien Giang. A total of 1310 transects were established perpendicularly to the baseline along the Tan Phu Dong District, with a 10 m spacing to ensure that each transect intercepted the shoreline only once to ensure exact calculation. Ngang Island was estimated automatically by the function in ArcGIS.

During the use of DSAS, the shoreline change envelope (SCE), net shoreline movement (NSM), linear regression rate of change (LRR), and end point rate (EPR) were used to determine the changes in the Tien Giang shoreline during the above periods.

The EPR equation is as follows (6):

Sr = (f0 − fy)/n,(6)

where

- Sr is the per year rate of shoreline change (m/year);
- f0 is the distance between the baseline and shoreline at the oldest date of a particular transect (m);
- fy is the distance between the baseline and shoreline on the most recent date on the same transect (m);
- n is the total number of years from the oldest date to the most recent date.

The equation for LRR is below (7).

L = at + b.(7)

where

- L represents the distance of the shore position from the baseline (m);
- t is the shoreline date interval (years);
- a is the slope of the fitted line (m/year) (i.e., the shoreline change rate—LRR);
- b is the y-intercept.

**Figure 2:**
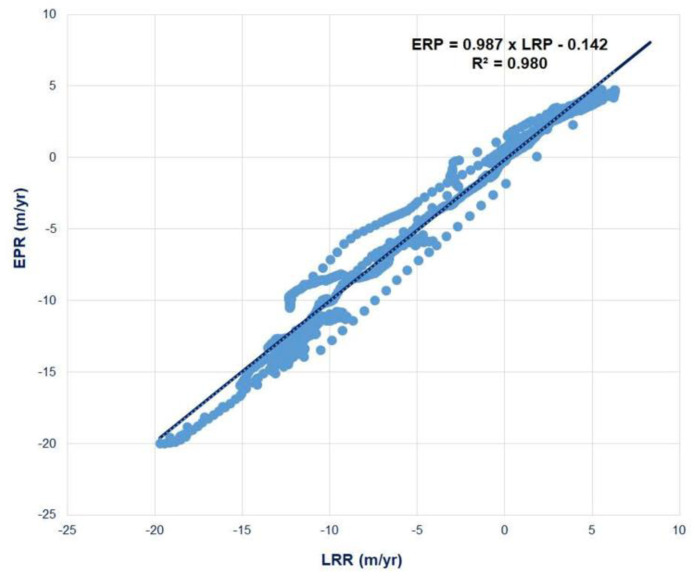
Correlations between the different statistical methods EPR vs. LRR in the Tan Phu Dong district, Tien Giang Province, Vietnam.

Both the LRR and EPR were used in this study because they provide small differences in the computed results. The SCE was estimated as the distance between the shoreline position and the baseline at each transect according to the latest (2022) and earliest (2000) sets of images, as pointed out previously [25,29,30,31,32]. Excellent correlations were obtained between the two statistical methods, with a high R^2^ value of 0.980 for Tan Phu Dong, indicating that the shoreline change rates in the Tan Phu Dong District were consistent with those of the previous two methods (Figure 2).

The SCE equation is below (8).

Sd = df − dc,(8)

where

- Sd is the shoreline change distance (m);
- df is the distance between the baseline and farthest shoreline (m) at a particular transect;
- dc is the distance between the baseline and closest shoreline (m) along the same transect.

The NSM equation is as below (9).

Snm = f0 − fy(9)

where

- Snm is net the movement of the shoreline (m);
- f0 is the distance between the baseline and shoreline (m) on the oldest date of a particular transect;
- fy is the distance between the baseline and shoreline (m) on the youngest date of the same transect.

The second component of the study, conducted between May 2020 and May 2024, involved five field visits/ground truthing to the site and its shoreline. The ground truthing involved the collection of GPS data of Tien Giang HTBs and the selection of six random sites along the Tan Phu Dong coast (four sites within the HTBs and two outside Tien Giang HTBs) for monitoring in May 2020 (see Figure 1 for further information of the random sites and Tien Giang HTBs) (Figure 1 and Table 2).

The 2000–2022 vegetation cover data were subsequently classified into non-vegetation cover (intertidal mudflats and barren lands) and vegetation cover (low vegetation cover, medium vegetation cover, and high vegetation cover) (Table 3).

The monitoring started in May 2020 and was undertaken every six months. Three elements selected as the criteria for monitoring were fine-grained sediment, coarse-grained sediment, and mangrove growth. The selection of these criteria was based on the pursuit of the objectives of the study. The monitoring aimed to describe the status of the random sites and take photographs of non-vegetated and vegetated areas in order to determine topographic reference points, main geographical features, vegetation patterns, and recent occurrences (natural regeneration of mangrove species) for the EVI analysis, not to quantify these criteria at each monitoring site.

## 3. Results

### 3.1. The Vegetation Cover Between 2000 and 2022

**Figure 3:**
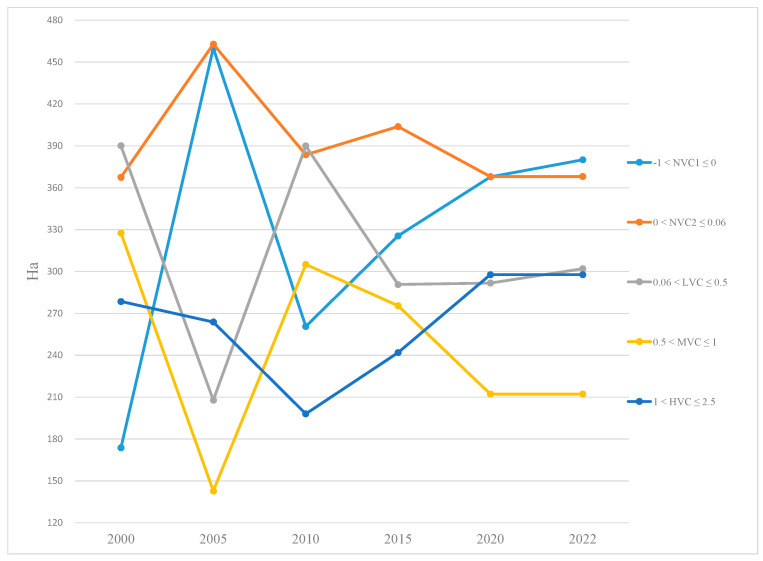
Vegetation cover changes at the study site between 2000 and 2022.

**Figure 4:**
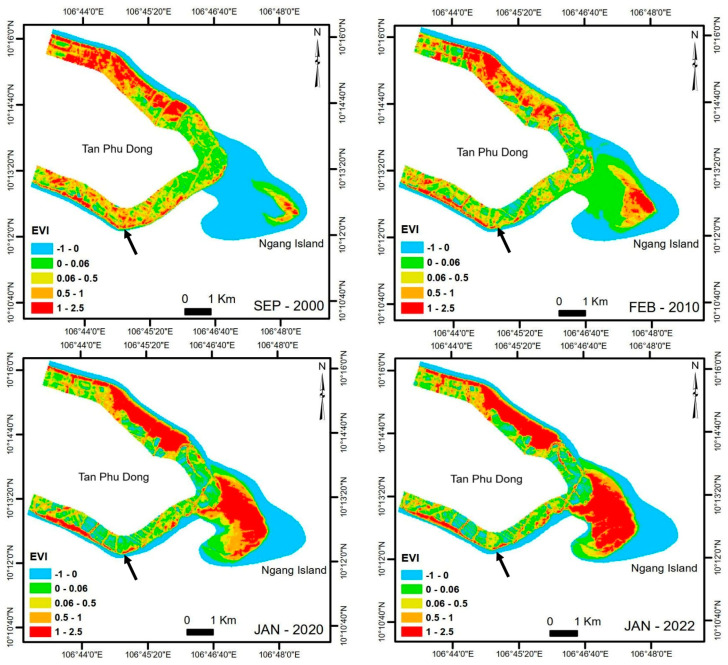
The vegetation cover and EVI values at the Tan Phu Dong coast, Tien Giang Province, Vietnam between 2000 and 2022. The black arrow shows the location of the study site.

The EVI analysis showed that by 2010, NVC1 and NVC2 had increased dramatically in 2005 (460 ha and 463 ha, respectively) and then decreased in 2010 (261 ha and 384 ha, respectively). While the LVC remained almost unchanged between 2000 and 2010, the MVC decreased from 328 ha in 2000 to 143 ha in 2005 before it reached 305 ha in 2010. The HVC constantly decreased from 278 ha in 2000 to 198 ha in 2010. By 2015, all the categories increased slightly except for LVC and MVC. LVC decreased from 390 ha in 2010 to 291 ha in 2015, while MVC decreased from 305 ha in 2010 to 275 ha in 2015. By 2020, all the categories decreased slightly except for NVC2 and MVC. NVC2 had decreased slightly from 404 ha in 2015 to 368 ha in 2015. The MVC decreased slightly from 275 ha in 2015 to 212 ha in 2020. NVC1, LVC, and HVC increased slightly from 2015 (326 ha, 291 ha, and 249 ha, respectively) to 2020 (368 ha, 292 ha, and 298 ha, respectively). By 2022, NVC2, MVC, and HVC remained unchanged, while NVC1 and LVC increased slightly from 368 ha and 292 ha in 2015, respectively, to 380 ha and 302 ha, respectively (Figure 3 and Figure 4).

### 3.2. The Shoreline Change Between 2000 and 2022

**Figure 5:**
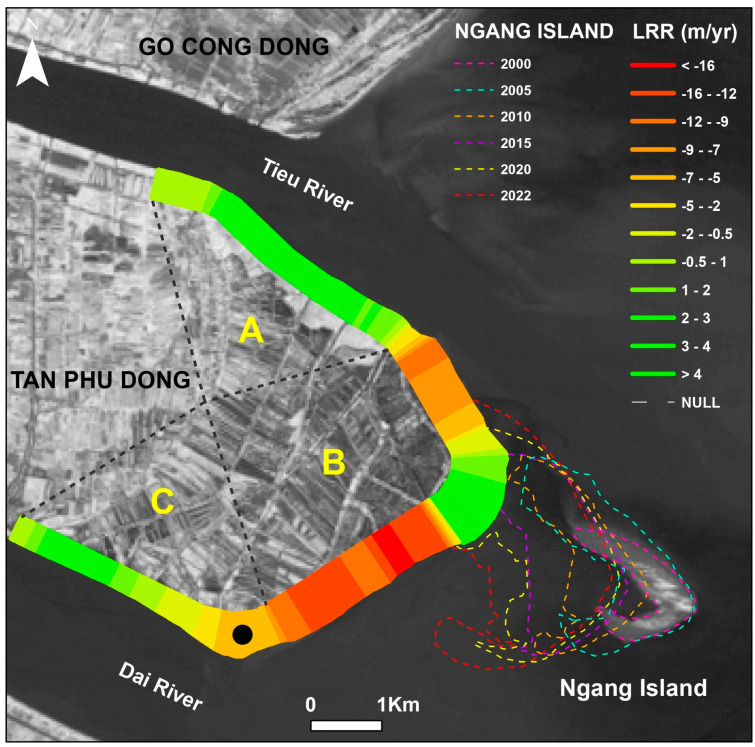
The shoreline changes in Tan Phu Dong District, Tien Giang Province between 2000 and 2022. The left image shows the short-term change rates in meters per year (LRR values) during the above periods [33]. The graph indicates long-term net shoreline changes in meters (SCE and 371 NSM values) and short-term change rates in meters per year (LRR and EPR values).

The DSAS indicated that Tien Giang HTBs were in slightly eroded areas, while areas located in northeast of Tien Giang HTBs experienced dramatic changes in the shoreline. Moreover, the southwest (river mouth area) area of HTBs was rather stable (Figure 5).

The Tan Phu Dong shoreline is quite complex and widely curved with two river areas. The complex and widely curved coasts made it quite difficult to calculate the rate of shoreline change. Therefore, the Tan Phu Dong coast was divided into three smaller areas (i.e., A, B, and C) that helped to constructed transects to accurately calculate the changes of the Tan Phu Dong shoreline. A–C and the dotted lines were part of the transact establishment and were used for calculating the changes in the Tan Phu Dong shoreline in this study. 

### 3.3. The Mangrove Growth

**Figure 6:**
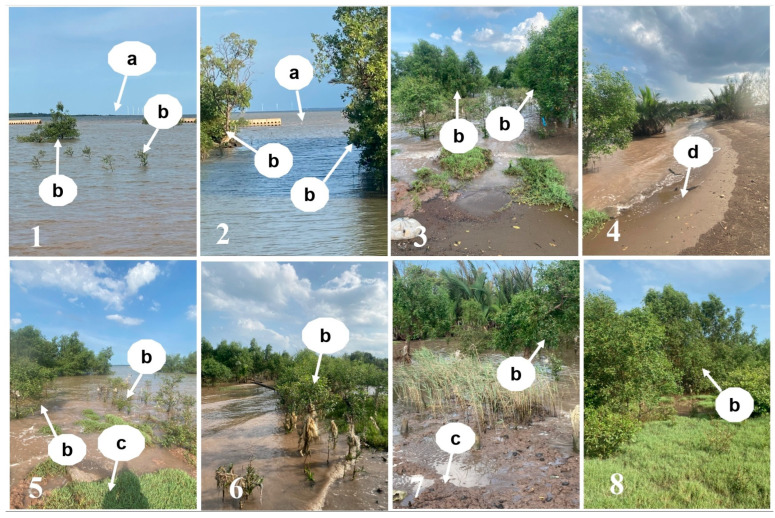
Natural growth of mangrove trees in the monitoring sites behind Tien Giang HTBs and two control sites. (**1**–**6**) Photos of the monitoring sites between Tien Giang HTBs and the shoreline, taken from the shore. (**7**,**8**) Photos of two control sites in the Dai river mouth areas, taken from the shore, showing the mangrove trees growing along the shore (see Figure 1, Figure 3 and Figure 6 for more information on the Tien Giang HTB deployment and the random and control sites). In the photo (1), (a) shows the 20 m space between two Tien Giang HTB sections; (b) indicates young *Bruguiera* trees growing behind HTBs. In the photo (2), (a) shows a 20 m space between two Tien Giang HTB sections; (b) is the fully grown *Bruguiera* trees behind HTBs along the shoreline. In the photos of 3,5–7, (b) shows a great number of young and fully grown trees of *Sonneratia* species, *Avicennia* sp, and *Bruguiera* species close to the shoreline and (c) are weeds (*Cyperus stoloniferus*) growing on clay soil. In the photo (4), (d) shows the sand accumulation along the shoreline. In the photo (8), (b) is fully grown trees of *Sonneratia* species, *Avicennia* sp., and *Bruguiera* species growing close to the shoreline and river mouth area, together with thick layers of weed species (*Cyperus stoloniferus*) growing on clay soil.

The monitoring revealed that there was a small number of young mangrove trees, mainly of the *Bruguiera* species, growing in small clusters in the gap between Tien Giang HTB section and the shoreline. There was also a low level of fine-grained and coarse-grained sediments at these monitoring sites behind Tien Giang HTBs over the entire period. Fully grown *Bruguiera* trees were also found in these monitoring sites behind Tien Giang HTBs. Meanwhile, two control sites experienced a high level of natural growth of mangrove species, including *Sonneratia*, *Avicennia*, and *Bruguiera* species in areas close to the shore and Tieu river mouth. *Sonneratia* sp. formed the majority, followed by *Bruguiera* sp., and *Avicennia* sp. These mangrove species grew from the shore. The monitoring also showed that remnants of eroded areas were still visible during low tides. Weeds, mainly *Cyperus stoloniferus*, grew strongly in brown clay soils (Table 4 and Figure 6).

## 4. Discussion

### 4.1. Tien Giang HTBs and Vegetation Cover Increase

The present study revealed that the vegetation cover has increased steadily since 2005, which was prior to the construction of Tien Giang HTBs. Vegetation cover in 2010 almost gained its highest coverage status in 2000 (Figure 3 and Figure 4). HVC increased dramatically between 2015 and 2020 while LVC and MVC decreased slightly between 2015 and 2020 (Figure 3 and Figure 4). Similarly, NVC1 and NVC2 fluctuated slightly during this period (Figure 3 and Figure 4). While MVC and HVC remained unchanged, LVC increased slightly between 2020 and 2022. Mangrove trees grew strongly in areas close to the shoreline and the estuarine areas (Figure 6). In addition, large areas of fully grown mangrove trees were found along the shoreline behind Tien Giang HTBs, particularly close to the river mouth areas (Figure 6 and Table 4). Weeds grew strongly on brown clay soil (Figure 6 and Table 4).

### 4.2. Mangrove Growth Process

The strong mangrove growth occurred partly due to the adaptation of mangrove species, with the dominance of *Sonneratia* and *Bruguiera* species to the site (estuarine areas) (two control sites). A previous study confirmed the occurrence of *Sonneratia* sp. in estuarine areas in many areas of the tropical areas [34]. A practical study showed that *Sonneratia* sp. can regenerate in well-protected or sheltered areas in the region [35].

*Bruguiera* species in the monitoring sites started growing in small clusters in 2000 (Table 4). In addition, weed species were growing strongly on remnants of eroded areas (brown clay soil) along the shore and coarse-grained sediment (mainly sand). The mangrove growth was possible particularly due to the existence of Ngang Island, which helped shelter the study site from the monsoon regimes (Figure 4). Ngang Island first appeared in approximately 2000 due to the gradual accumulation of sediment (NVC1) (Figure 4). The evolution of Ngang Island was fully reported [33]. This sediment may have been discharged from the Tieu River mouth (see Figure 5 for the location of the Tieu River mouth). Similar sedimentation processes have been reported in the region in previous studies [36,37,38,39]. The strong growth of the *Bruguiera* species and weed growth (Figure 6 and Table 4) indicate that the site has gradually stabilized, with increased vegetation cover since 2010 due to the existence of Ngang Island, which happened before Tien Giang HTBs were constructed. This means that mangrove species grew stronger and diverse under favorable conditions, and that there is no need to plant mangrove seedlings under similar conditions, which was confirmed by the previous studies [40,41].

### 4.3. Tien Giang HTBs in Mangrove Protection

Offshore breakwaters (emergent detached and submerged offshore structures) are increasingly being constructed to control coastal erosion in Vietnam [16,42,43,44] and have been increasingly used throughout Southeast Asia [45]. To date, previous studies have shown the effectiveness of offshore structures in dissipating the energy of strong incident waves [12,13,46,47]. In this study, the DSAS showed that Tien Giang HTBs were constructed in slightly eroded areas and in estuarine areas (Figure 5), after they had been numerically tested. Three different mangrove species (*Sonneratia*, *Avicennia*, and *Bruguiera* species) grew in estuarine areas, while only one species (*Bruguiera* species) grew in small clusters behind Tien Giang HTBs (Figure 4 and Figure 6 and Table 4). This means that numerical testing using wave parameters is insufficient for improving the effectiveness of HTBs in promoting mangrove growth or increasing vegetation cover. In addition, the current Tien Giang HTB location (slightly eroded areas and estuarine area) was not solid evidence that Tien Giang HTBs drove increases in vegetation cover or the natural regeneration of mangrove species in the study area. Therefore, the morpho-hydrodynamic characteristics of the site must be sufficiently considered during the design of HTBs, as previously recommended [48,49], to maximize their effectiveness. Likewise, HTBs should be constructed in areas other than stable or estuarine areas to test their ability to increase vegetation cover. Likewise, increases in vegetation cover and the natural regeneration of local mangrove forests appear not to have been the focus of the technical design of these structures, which were primarily constructed to dissipate wave energy and reduce shoreline erosion.

### 4.4. Limitations of This Study

The main objective of this study was to investigate the relationship between Tien Giang HTBs and vegetation cover increase. This was undertaken mainly using EVI calculations, DSAS, and field observations. The process underlying how Ngang Island supported natural regeneration and secondary succession of these mangroves and protected vegetation cover within the study site warrants further study. In addition, the reason why the LVC, MVC, and HVC decreased dramatically between 2000 and 2005 needs to be further studied. A targeted investigation of local morpho-hydrodynamic characteristics combined with appropriate monitoring could help to better understand the interactions between physical and ecological processes in this region of the Mekong Delta. If possible, morpho hydrodynamic characteristics should be examined in detail to better understand these processes. In addition, appropriate monitoring and evaluation should be implemented to better understand ecological processes.

## 5. Conclusions

The findings of this study on the relationship between Tien Giang HTBs and mangrove vegetation cover in Go Cong, Tien Giang, Vietnam, suggest that Tien Giang HTBs have yet to increase mangrove vegetation cover or promote any mangrove growth three years after their construction. The vegetation cover, dominated by *Sonneratia*, *Avicennia*, and *Bruguiera* species, steadily increased in parallel with a high level of mangrove growth, particularly with large areas of fully grown mangrove trees. An increase in vegetation cover, along with natural regeneration, occurred even before Tien Giang HTBs were constructed. This could be partly due to the existence of Ngang Island, which provided substantial shelter to the study site and the construction of these structures. In addition, Tien Giang HTBs were constructed in a rather stable area (both slightly eroded and estuarine area), and therefore, have yet to provide any noticeable benefits for mangrove regeneration three to five years after their construction.

This study shows that mangrove forests grew strongly in sheltered areas or estuarine areas, which were dominated by fine-grained sediments and that Tien Giang HTBs supported mangrove growth. There are, therefore, two options available to coastal managers. The first option is to construct HTBs in areas other than stable or estuarine areas to test their ability to increase vegetation cover. The second option is to apply a gradual expansion method [41] to ensure strong protection of mangrove forests and the Tien Giang coast. HTBs should be constructed in natural mangrove succession and/or strong mangrove growth as a first stage toward expanding existing mangrove areas along the coast. The second option is more suitable for those who are reluctant to adopt the first option as a solution.

## Figures and Tables

**Table 1:** Satellite images were retrieved and analyzed in this study.

Year	Path	Row	Acquisition Date	Satellite Image	Cloud Cover	Cloud Cover Land	Spatial Resolution (m)
2000	125	053	27 September 2000	Landsat-5 (TM)	15	13	30
2005	125	053	13 February 2005	Landsat-5 (TM)	12	12	
2010	125	053	27 February 2010	Landsat-5 (TM)	20	23	
2015	125	053	9 February 2015	Landsat-8 (OLI)	2.86	3.37	
2020	125	053	6 January 2020	Landsat-8 (OLI)	2.28	2.18	
2022	125	053	11 January 2022	Landsat-8 (OLI)	1.43	2.43	

**Table 2:** Locations and types of Tien Giang HTBs in Tan Phu Dong District, Tien Giang Province.

Section	Type	GPS Coordinates		Length (m)
		Start Point	End Point	
0		10°12′17.48″ N; 106°45′11.48″ E	10°12′16.22″ N; 106°45′13.63″ E	79
1B	HTB—Type A	10°12′16.23″ N; 106°45′13.84″ E	10°12′18.59″ N; 106°45′17.20″ E	127
2B		10°12′18.87″ N; 106°45′17.82″ E	10°12′21.38″ N; 106°45′21.48″ E	136
3B		10°12′21.74″ N; 106°45′22.04″ E	10°12′24.24″ N; 106°45′25.73″ E	135
4C	HTB—Type B	10°12′24.60″ N; 106°45′26.37″ E	10°12′26.99″ N; 106°45′30.00″ E	135
5C		10°12′27.37″ N; 106°45′30.56″ E	10°12′29.81″ N; 106°45′34.29″ E	135
6C		10°12′30.17″ N; 106°45′34.80″ E	10°12′32.57″ N; 106°45′38.45″ E	135
7C		10°12′32.94″ N; 106°45′39.00″ E	10°12′35.40″ N; 106°45′42.78″ E	135
8C		10°12′35.79″ N; 106°45′43.32″ E	10°12′37.96″ N; 106°45′47.05″ E	135
9C		10°12′38.27″ N; 106°45′47.71″ E	10°12′39.64″ N; 106°45′51.79″ E	135
10C		10°12′39.92″ N; 106°45′52.47″ E	10°12′41.34″ N; 106°45′56.59″ E	135

**Table 3:** Levels of vegetation cover and corresponding EVI values used in this study.

No.	Levels of Vegetation Cover	EVI Values
1	Non-vegetation cover—Intertidal mudflats—NVC 1	−1 < EVI ≤ 0
2	Non-vegetation cover—Barren lands—NVC 2	0 < EVI ≤ 0.06
3	Low vegetation cover—LVC	0.06 < EVI ≤ 0.5
4	Medium vegetation cover—MVC	0.5 < EVI ≤ 1
5	High vegetation cover—HVC	1 < EVI ≤ 2.5

**Table 4:** Status of the monitoring sites as of May 2024.

Monitoring Sites				
Location	Mangrove Forest	Seasonal Inundation	Sediment Accumulation	Remarks
The Monitoring Sites Behind the HTBs				
1	•	•	•	*Bruguiera* mature trees grew in clusters in seasonally inundated areas with fine-grained sediment
2	•	•	•	*Bruguiera* mature trees grew in clusters in seasonally inundated areas with fine-grained sediment
3	•	•	•	*Bruguiera* mature trees grew in clusters in seasonally inundated areas with fine-grained sediment
4	•	•	•	*Bruguiera* mature trees grew in clusters in seasonally inundated areas with fine-grained sediment
The monitoring sites outside HTBs				
5	•	•	•	✓ *Bruguiera Sonneratia* and *Avicennia mature* trees grew in clusters in estuarine areas. ✓ Coarse-grained sediment (mainly sand) found along the shore. ✓ Weed species (*Cyperus stoloniferus*) growing on remnants of eroded area (mainly brown clay soil).
6	•	•	•	✓ *Bruguiera* mature trees grew in clusters in seasonally inundated areas with fine-grained sediment ✓ Coarse-grained sediment (mainly sand) found along the shore. ✓ Weed species (*Cyperus stoloniferus)* growing on remnants of eroded area (mainly brown clay soil).

## Data Availability

Data available in a publicly accessible repository.
